# Curcumin: A dietary phytochemical for boosting exercise performance and recovery

**DOI:** 10.1002/fsn3.2983

**Published:** 2022-07-18

**Authors:** Mohammad Nosrati‐Oskouie, Nazanin Sadat Aghili‐Moghaddam, Omid Mohammad Tavakoli‐Rouzbehani, Tannaz Jamialahmadi, Thomas P. Johnston, Amirhossein Sahebkar

**Affiliations:** ^1^ Department of Clinical Nutrition and Dietetics, Faculty of Nutrition Sciences and Food Technology, National Nutrition and Food Technology Research Institute Shahid Beheshti University of Medical Sciences Tehran Iran; ^2^ Nutrition and Endocrine Research Center, Research Institute for Endocrine Sciences Shahid Beheshti University of Medical Sciences Tehran Iran; ^3^ Department of Nutrition, Faculty of Medicine Mashhad University of Medical Sciences Mashhad Iran; ^4^ Department of Clinical Nutrition, Faculty of Nutrition and Food Science Tabriz University of Medical Sciences Tabriz Iran; ^5^ Biotechnology Research Center Mashhad University of Medical Sciences Mashhad Iran; ^6^ Division of Pharmacology and Pharmaceutical Science, School of Pharmacy University of Missouri‐Kansas City Kansas City Missouri USA; ^7^ Applied Biomedical Research Center Mashhad University of Medical Sciences Mashhad Iran; ^8^ Biotechnology Research Center, Pharmaceutical Technology Institute Mashhad University of Medical Sciences Mashhad Iran; ^9^ School of Medicine The University of Western Australia Perth Western Australia Australia; ^10^ Department of Biotechnology, School of Pharmacy Mashhad University of Medical Sciences Mashhad Iran

**Keywords:** athletes, curcumin, exercise, metabolism

## Abstract

Curcumin, as the main natural compound in the turmeric plant (*Curcuma longa*), is a yellowish polyphenol that has been used traditionally in Asian countries as a medicinal herb for various types of disease and pathological conditions caused by inflammation and oxidative stress. In the present review, we conducted a comprehensive literature search for evidence that shows the effect of curcumin on factors influencing exercise performance, including muscle damage, muscle soreness, inflammation, and oxidative stress. During exercise, reactive oxygen species and inflammation are increased. Thus, if there is no balance between endogenous and exogenous antioxidants and increases in oxidative stress and inflammation, which is important for maintaining redox homeostasis in skeletal muscle, it can lead to muscle soreness and muscle damage and ultimately result in reduced exercise performance. Due to the anti‐oxidant and anti‐inflammatory properties of curcumin, it can increase exercise performance and decrease exercise‐induced muscle soreness and muscle damage. It appears that curcumin supplementation can have positive effects on exercise performance and recovery, muscle damage and pain, inflammation, and oxidative stress. However, there is still a need to precisely evaluate factors to more accurately assess/quantify the beneficial therapeutic effects of curcumin with regard to enhancing exercise performance and recovery.

## Graphical abstract

Curcumin, by functioning as an antioxidant, may reduce oxidative stress to cells, and especially mitochondria, in the long‐term, as well as positively impact exercise endurance, strength, and recovery to improve overall health.

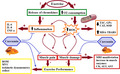

## 1 INTRODUCTION

### 1.1 Exercise and its adverse effects

Exercise is generally divided into two categories: aerobic/endurance and power/strength (Hughes et al., 2018). Endurance exercises, such as running, swimming, and cycling, are classically performed against a relatively low load over a long duration, whereas strength exercises, like bodybuilding, are performed against a relatively high load for a short period (Hughes et al., 2018; Morici et al., 2016). However, a single type of each exercise is rare, and exercises are usually a mixture of both types, and termed concurrent exercise. Accumulation of hydrogen ion (i.e., lactic acidosis) and heat (i.e., hyperthermia) are the main adverse effects of extreme and prolonged oxidative metabolism associated with endurance exercise (Coyle, 1999). In fact, oxygen‐containing free radicals such as the hydroxyl ions, superoxide, hydroperoxyl, and lipid peroxyl are the compounds generated after exercise. Additionally, intense exercise training or muscle contractions during high‐intensity exercise can trigger an inflammatory response with an increase in expression of interleukin 6 (IL‐6), interleukin 8 (IL‐8), tumor necrosis factor‐α (TNF‐α), and the production of reactive oxygen species (ROS) (Tanabe, Chino, Ohnishi, et al., 2019). A balance between ROS production and antioxidant enzyme expression is important to sustain muscle redox homeostasis (Accattato et al., 2017). Whenever the production of these radicals and the inflammatory response exceeds the capacity of the tissue, secondary muscle damage can occur. Evidence suggests that this damage can lead to fatigue, muscle soreness, onset of muscle damage, decreased muscle strength and range of motion (ROM), and an increase in creatine kinase (CK) activity as determined in the blood (Basham et al., 2019; Vincent, 2004). The muscle damage and inflammatory responses may impact athletic performance.

### 1.2 Curcumin

Curcumin is a yellowish polyphenol that has traditionally been used in Asian countries as a medicinal herb for various pathological conditions such as dermatologic diseases, infection, and reducing inflammation, stress, and depression (Debjit Bhowmik et al., 2009; Kocaadam & Şanlier, 2017). It is the main natural compound in the turmeric plant (*Curcuma longa*), which is wildly cultivated in Indonesia, China, and India (Hewlings & Kalman, 2017). Although studies conducted on the bioavailability and pharmacokinetics of curcumin have shown low oral absorption and rapid removal (clearance) from the body, it is generally recognized as safe by the United States Food and Drug Administration, making this natural compound suitable for the treatment of various maladies (Bagherniya et al., 2018; Farhood et al., 2019; Nosrati‐Oskouie et al., 2021; Panahi et al., 2014; Panahi et al., 2017; Parsamanesh et al., 2018; Rauf et al., 2018; Sahebkar, 2010; Shakeri et al., 2019; Afshari et al., 2021; Gorabi et al., 2019). The beneficial therapeutic properties of curcumin have been associated with its chemical structure, which enable it to interact with molecules inside the cell and mediate a variety of biological outcomes such as anti‐oxidant and anti‐inflammatory effects (Stanić, 2017).

### 1.3 Antioxidant properties of curcumin

Curcumin can alter several indices and biomarkers of oxidative stress in different physiological and pathophysiological conditions. Either the phenolic OH groups or the CH2 group of the β‐diketone moiety, may be responsible for the ROS scavenging activity of curcumin (Rauf et al., 2018; Takahashi et al., 2014). Curcumin can scavenge many different kinds of free radicals such as ROS and reactive nitrogen species (RNS). It effects on free radicals is carried out by several mechanisms. For example, it can inhibit the activity of xanthine hydrogenase/oxidase and lipoxygenase/cyclooxygenase, which are ROS‐generating enzymes. Additionally, it can modulate enzymes in the neutralization of free radicals such as superoxide dismutase (SOD), glutathione peroxidase (GPx), and catalase by blocking the PI3K/Akt/NF‐κB signaling pathway (Hewlings & Kalman, 2017; Li et al., 2018; Lin et al., 2007). Also, SOD gene expression was found to be suppressed by bile acid, which was reversed by curcumin treatment (Bower et al., 2010).

### 1.4 Anti‐inflammatory properties of curcumin

The pathological processes of oxidative stress and inflammation are closely related to each other. An intercellular signaling cascade can be initiated by several ROS, which leads to an increase in pro‐inflammatory gene expression (e.g., interleukin‐8, monocyte chemoattractant protein‐1, cyclooxygenase‐2, and epidermal growth factor receptor) (Rahman et al., 2004). TNF‐α is the primary mediator of inflammation in numerous disease states. Curcumin can block the activation pathways of TNF‐α and nuclear factor kappa‐light‐chain‐enhancer of activated B cells (NF‐kB), which are activated by ROS (Kelany et al., 2017; Powers & Jackson, 2008; Sahebkar et al., 2016; Zhong et al., 2016). Moreover, studies have suggested that curcumin can alleviate inflammation by acting through the Nrf2 (nuclear factor erythroid 2 [NF‐E2]‐related factor 2 [Nrf2])‐Keap1 (Kelch‐like erythroid cell‐derived protein with CNC homology [ECH]‐associated protein 1) pathway (Anthwal et al., 2014; Gupta et al., 2014), and Nrf2 may be associated with mitogen‐activated protein kinase (MAPK), NF‐kB, phosphoinositide 3‐kinase (PI3K), and protein kinase C (PKC) pathways (Cordero‐Herrera et al., 2015; He et al., 2015).

### 1.5 Curcumin and exercise

Alleviating inflammatory and oxidative stress could be a potential mechanism to accelerate the adaptation to prolonged exercise (Powers & Jackson, 2008). Among antioxidant agents, curcumin, the main component of turmeric, is one of the most cost‐effective, readily available, and safe natural products (White et al., 2019). Several studies have reported the safety and efficacy of this natural compound in various diseases such as cancer, arthritis, metabolic syndrome, anxiety, depression, and other medical conditions caused by inflammation and oxidative stress (Daily et al., 2016; Farzaei et al., 2018; Hewlings & Kalman, 2017; Kelany et al., 2017; Ng et al., 2017; Oskouie et al., 2019). Previous studies have also reported that curcumin possesses not only potent activity as an antioxidant and anti‐inflammatory agent, but also exerts beneficial therapeutic effects on wound healing (Tejada et al., 2016; Zhong et al., 2016). The anti‐inflammatory properties of curcumin are derived from the capacity of this polyphenol to negatively regulate pro‐inflammatory cytokines (Bianconi et al., 2018; Derosa et al., 2016) including IL‐1 and TNF‐α as shown in a meta‐analysis of randomized controlled trials (Gorabi et al., 2021). Curcumin is thought to be effective in reducing inflammation through the NF‐κB and IκB kinase signaling pathways (Shishodia et al., 2005). Moreover, as it pertains to curcumin's anti‐oxidant effect (Kelany et al., 2017; Powers & Jackson, 2008; Sahebkar et al., 2016; Zhong et al., 2016), other studies have reported a decrease in CK activity, inflammatory biomarkers such as IL‐8 and TNF‐α, and improvement in muscle soreness (Tanabe, Chino, Ohnishi, et al., 2019). However, the exact effects of curcumin on the inflammation, oxidative stress, and muscle damage that result from exercise, as well as the optimal dose, frequency, and duration of curcumin consumption/supplementation, is still unclear. Therefore, this review was undertaken to summarize the effect of curcumin supplementation on exercise and muscle performance in an attempt to better define its therapeutic benefits.

### 1.6 Effect of curcumin on oxidative stress, inflammation, and muscle damage induced by exercise

#### 1.6.1 Preclinical data

Several in vivo studies have demonstrated that dietary curcumin supplementation leads to an improvement in indicators of muscle damage (such as CK, AST, ALT), inflammation (such as IL‐6 and TNF‐α), and oxidative stress (such as SOD, HO‐1) induced by exercise. Furthermore, it has been reported that curcumin administration can increase exercise performance due to its anti‐inflammatory and antioxidant properties, as well as through mitochondrial biogenesis (Sahin et al., 2016). In a study by Sahin et al., the antioxidant activity of curcumin was investigated in rats that were subjected to exhaustive exercise. Their results showed that a water‐soluble curcumin formulation (20% curcuminoids) at a daily dose of 100 mg/kg for 6 weeks significantly increased the time to exhaustion, muscle antioxidant enzymes, and proteins including superoxide dismutase (SOD), GPx, glutathione, catalase, NF‐ҡB, inhibitors of kappa B, and thioredoxin‐1 in curcumin‐treated rats when compared to controls. However, these same authors showed that curcumin, at the dose mentioned above, also significantly decreased lactate, malondialdehyde (MDA), and heat shock protein 70 in curcumin‐treated rats versus controls. Taken together, this study reported that curcumin treatment increased Nrf2, sirtuin 1, hemeoxygenase‐1 (HO‐1), and peroxisome proliferator‐activated receptor‐gamma coactivator 1‐α (PGC‐1α) (Sahin et al., 2016). Nrf2, as the major regulator of the antioxidant defense system, may play a substantial role in the outcome of exercise‐induced oxidative stress (Done & Traustadottir, 2016). Hemeoxygenase‐1, PGC‐1α (through its interaction with Nrf2), and sirtuin 1 via PGC‐1α deacetylation have all been suggested to function as potential regulators of mitochondrial biogenesis and enhance the capacity of exercise endurance (Piantadosi et al., 2008; Suwa et al., 2008). To confirm this hypothesis, 4 weeks of daily intraperitoneal injection of curcumin in rats with, or without, exercise endurance training, resulted in an up‐regulation in protein expression of sirtuin 1, mitochondrial markers (cytochrome c oxidase subunit IV and oxidative phosphorylation subunits), mitochondrial DNA copy number, and citrate synthase activity in gastrocnemius and soleus muscles. Additionally, this study demonstrated an increase in the PGC‐1α deacetylation and phosphorylation of adenosine monophosphate‐activated protein kinase (indirectly as a sirtuin 1 inducer and PGC‐1α activator). Consequently, curcumin increased the nicotinamide adenine dinucleotide (NAD) to NAD^+^ hydrogen ratio, which increased mitochondrial biogenesis in skeletal muscle (Hamidie et al., 2015). Further evidence in the literature has indicated that apoptosis can occur after exercise, and it has been suggested that mitochondria may play a key role in regulating the apoptosis (Phaneuf & Leeuwenburgh, 2001). Using rats that were made to exercise by “running‐to‐exhaustion” on a treadmill, Jiliang et al. reported a decrease in anti‐apoptotic protein of Bcl‐2 expression and an increase in apoptotic protein of Bax expression, which consequently gave rise to an increase in the Bax/Bcl‐2 ratio. Additionally, using immunohistochemical assays of pro‐ and anti‐apoptotic proteins, these same authors demonstrated increased expression of Bcl‐2, but decreased expression of Bax, and consequently, a reduction in the Bax/Bcl‐2 ratio, in rats that received 75 mg/kg of curcumin daily by oral gavage for 14 days when compared to these same corresponding measurements obtained for rats that underwent exhaustive exercise (Fu et al., 2010). Moreover, Wafi et al. (2018) studied changes in exercise performance and indicators of oxidative stress in both sham mice and mice with experimentally‐induced heart failure with, and without, curcumin administration. These authors found that curcumin administered subcutaneously for 8 weeks using an osmotic minipump increased their running distance (time to fatigue), as well as the expression of Nrf2, SOD, and HO‐1.

In a study conducted by Davis et al., the anti‐inflammatory effect, as well as any change in the extent of muscle damage produced by dietary curcumin administration, was evaluated using uphill and downhill running in mice. These authors reported that curcumin powder, at a daily dose of 10 mg in the diet for 3 days prior to the exercise, reduced exhaustion, increased the time and distance of running, and suppressed elevations in soleus muscle concentrations of TNF‐α, IL‐6, IL‐1β, and plasma CK levels following downhill running only (Davis et al., 2007). Finally, Huang et al. (2015) reported that oral administration of varying doses of curcumin to mice for 28 days prior to swimming experiments increased the time to exhaustion and muscle glycogen levels (as a source of energy during exercise) and reduced biochemical indicators related to fatigue and tissue damage including lactate, ammonia, glucose, blood urea nitrogen, CK, aspartate transaminase (AST), and alanine transaminase (ALT) following swimming.

**FIGURE 1:**
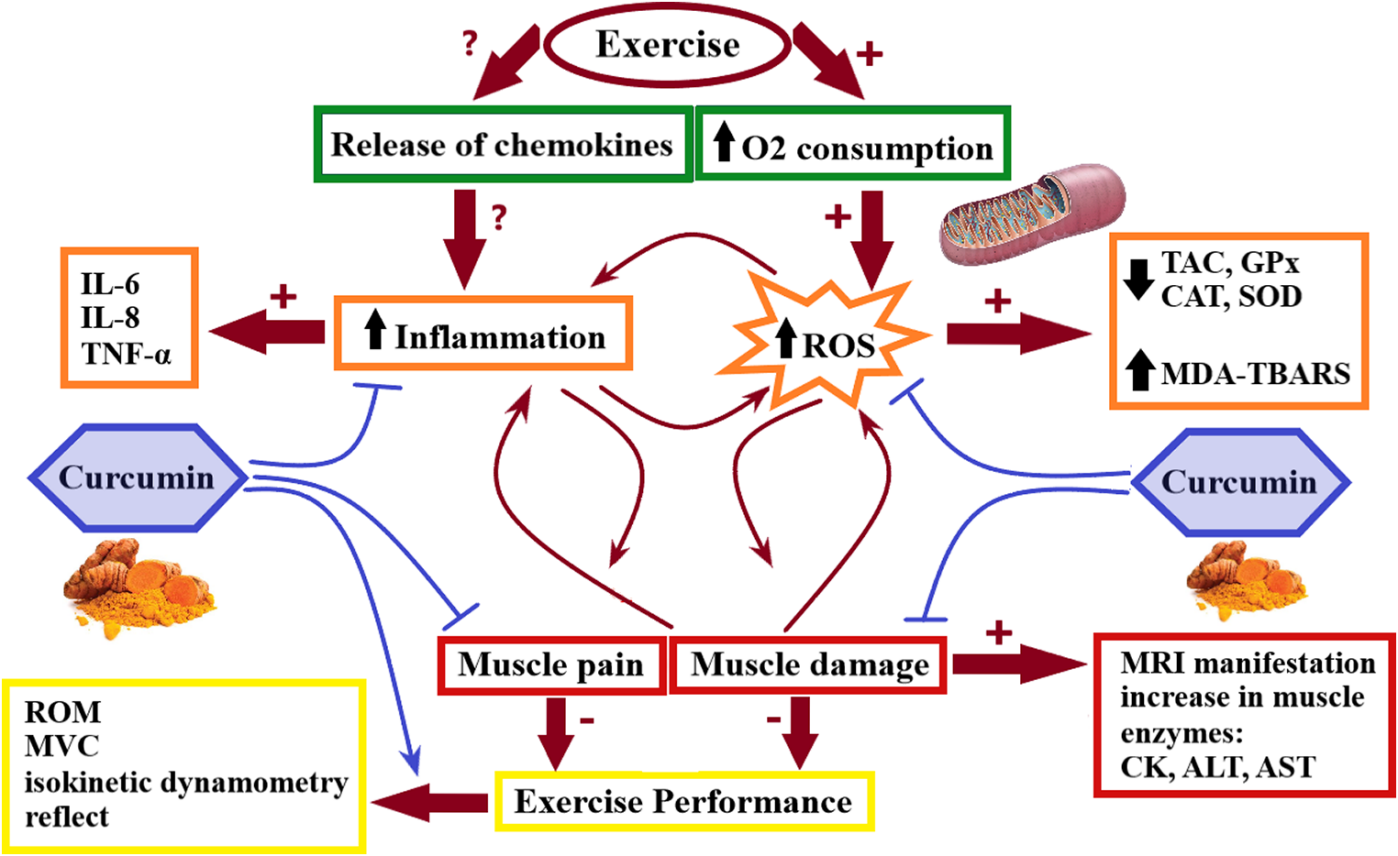
Schematic representations of the curcumin effect on the muscle damage and muscle soreness induced by exercise through anti‐inflammatory and anti‐oxidant properties that may eventually lead to improvement in exercise performance. + indicates an elevation, − indicates a reduction; ROS, reactive oxygen species; TAC, total antioxidant capacity; SOD, superoxide dismutase; GPx, glutathione peroxidase; CAT, catalase; CK, creatine kinase; ALT, alanine transaminase; AST, aspartate transaminase; MDA, malondialdehyde; TBARS, thiobarbituric acid‐reactive substance; MRI, magnetic resonance imaging; TNF‐α, tumor necrosis factor‐α; IL‐6, Interleukin 6; MVC, maximal voluntary contraction; ROM, range of motion.

In vivo studies that have evaluated the effect of curcumin on exercise performance have shown that curcumin can potentially improve performance by decreasing inflammation, oxidative stress, and muscle damage, and increasing mitochondria biogenesis and function, which subsequently results in an anti‐apoptotic effect (Figure 1). Major findings regarding the role of curcumin administration on exercise performance in vivo are summarized in Table 1.

**TABLE 1:** Characteristics of preclinical studies that evaluated the effect of curcumin supplementation on exercise performance

Author	Animal model	Dosage and periods	Treatments	Results
Sahin et al. (2016)	Male Wistar rats; n = 7 per group	20 mg curcumin daily; 6 weeks	Group 1: control Group 2: curcumin Group 3: exercise Group 4: curcumin + exercise	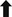 Run to exhaustion, SOD, GPx, GSH, CAT, and 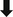 lactate, MDA, NF‐ҡB, HSP70 in group 2 and 4. 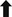 I‐κB, TRX‐1, SIRT1, PGC‐1α, Nrf2, HO‐1, and GLUT4 in group 2 vs others.
Davis et al. (2007)	Male mice; −	10 mg curcumin with diet; 3 days before trial	Group 1: downhill placebo Group 2: downhill curcumin Group 3: uphill placebo Group 4: uphill curcumin	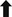 Time to exhaustion at 48 and 72 h, time and distance of running 24 h after test in group 2 vs, group 1. 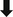 CK activity, muscle IL‐1β, IL‐6, and TNF‐α in group 2 vs group 1. Curcumin did not affect the uphill runner.
Huang et al. (2015)	Male ICR mice; *n* = 10 per group	12.3, 24.6, and 61.5 ml/kg curcumin daily; 28 days before trial	Group 1: vehicle Group 2: curcumin at 12.3 ml/kg Group 3: curcumin at 24.6 ml/kg Group 4: curcumin at 61.5 ml/kg	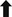 Time to exhaustion by 1.98‐, 2.17‐ and 2.22‐fold in group 1, 2, and 3 vs group 1 dose‐dependently. 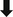 Serum lactate, ammonia, glucose, BUN, CK, AST, and ALT in group 1, 2, and 3 vs group 1 dose‐dependently. 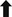 Muscle glycogen, but not liver, were higher in group 1, 2, and 3 vs group 1 dose‐dependently.
Wafi et al. (2018)	Male C57BL/6 mice; *n* = 40	50 mg/Kg curcumin daily; 8 weeks	Group 1: Sham‐Vehicle (*n* = 8) Group 2: Sham‐Curcumin (*n* = 9) Group 3: Heart Failure‐Vehicle (*n* = 12) Group 4: Heart failure‐Curcumin (*n* = 11)	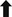 Time to exhaustion in group 2 vs. baseline and group 1. 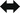 Distance and speed in group 4 vs baseline. 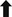 Nrf2, HO‐1, and SOD2 expression in group 2 and 4 vs group 1 and 3. 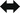 Keap1 and α‐Tubulin expression in group 2 and 4 vs group 1 and 3.
Fu et al. (2010)	Male Wistar rats; *n* = 8 per group	75 mg/kg curcumin daily; 14 days	Group 1: normal control Group 2: exercise control Group 3: curcumin	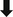 Bax, Bax/Bcl‐2, and 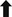 Bcl‐2 expression in group 3 vs group 2. 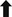 Bax and Bax/Bcl‐2 in group 3 vs group 1.
Hamidie et al. (2015)	Male Wistar rats; *n* = 6 per group	50 and 100 mg/kg curcumin daily; 28 days	Group 1: control Group 2: 50 mg/kg/day curcumin Group 3: 100 mg/kg/day curcumin Group 4: endurance training Group 5: 50 mg/kg/day curcumin + endurance training Group 6: 100 mg/kg/day curcumin + endurance training	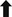 COX‐IV, OXPHOS subunits, mitochondrial DNA copy number, SIRT1, CS activity, AMPK phosphorylation, NAD+/NADH ratio, and PGC‐1α deacetylation in combination curcumin and training. 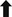 cAMP and PKA (phosphorylation CREB and LKB‐1) in curcumin and training groups.

*Note*: The up arrows indicate the elevation, the down arrows indicate the reduction, and the Left–Right Arrow indicate no change.

Abbreviations: AMPK, adenosine monophosphate‐activated protein kinase; ALT, alanine transaminase; AST, aspartate transaminase; CAT, catalase;CK, creatine kinase; COX‐IV, cytochrome c oxidase subunit IV; CS, citrate synthase; CREB, cAMP response element binding protein;cAMP, cyclic adenosine monophosphate; GLUT4, glucose transporter 4; GPx, glutathione peroxidase;GSH, glutathione; HO‐1, hemeoxygenase‐1; HSP70, heat shock protein 70; I‐κB, inhibitors of kappa B; IL‐6, Interleukin 6; Keap1, Kelch‐like ECH‐associated protein 1; LKB‐1, liver kinase B1;SOD, superoxide dismutase; MDA, malondialdehyde; NF‐kB, nuclear factor kappa‐light‐chain‐enhancer of activated B cells; Nrf2, nuclear factor (erythroid‐derived 2)‐like 2; NAD, nicotinamide adenine dinucleotide; NADH, nicotinamide adenine dinucleotide hydrogen; OXPHOS, oxidative phosphorylation; PGC‐1α, peroxisome proliferator‐activated receptor gamma coactivator 1‐alpha; PKA, protein kinase A;SIRT1, sirtuin 1; TRX‐1, thioredoxin‐1; TNF‐α, tumor necrosis factor‐α;.

#### 1.6.2 Clinical data

Exercise performance and heath may be affected by inflammation and oxidative stress, which can be caused by an imbalance between ROS, RNS, and endogenous antioxidants. Several clinical trials have investigated the efficacy of curcumin in reducing muscle damage and soreness induced by exercise via a reduction in oxidative stress and inflammation. The characteristics of these clinical trials are summarized in Table 2.

**TABLE 2:** Characteristics of clinical studies that evaluated the effect of curcumin supplementation on exercise performance

Author	Study design and duration	Participants (gender, age, *n*, condition)	Type of activity	Treatments	Results
Jäger et al. (2019)	RCT, DB; 8 weeks	Both, 21, 63, healthy and physically active	Downhill run	Group1: 50 mg of curcuminoids Group 2: 200 mg of curcuminoids Group 3: placebo	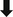 Isokinetic peak extension torque in group 1and 3. 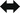 Isokinetic peak extension torque in group 2. 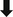 Isokinetic peak flexion torque and power in group 1. 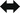 Isokinetic peak flexion torque and power in group 2 and 3. 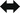 Isokinetic extension power and isometric average peak torque in group 1, 2, and 3. 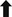 soreness in all the groups vs. baseline.
Tanabe et al. (2015)	RCT, SB, CO; −	Men, 24, 14, Young men without any regular resistance training for 1 year	Eccentric exercise of the elbow flexors	Group 1: 300 mg daily (150 mg 1 h before and 150 mg 12 h after exercise) Group 2: placebo	MVC torque reduced fewer, recovery was faster and peak serum CK activity was fewer in group 1 vs group 2. 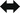 IL‐6, TNF‐α, ROM, and VAS‐muscle soreness between two groups.
Tanabe, Chino, Sagayama, et al. (2019)	RCT, SB; 7 days before and 4 days after exercise	Men, 29, 24, healthy	Eccentric exercise of the elbow flexors	Group 1: 180 mg/day (90 mg twice daily after breakfast and dinner) of curcumin before exercise for 7 days Group 2: 180 mg/day (90 mg twice daily after breakfast and dinner) curcumin after exercise for 4 days Group 3: placebo after breakfast and dinner for 4 days after exercise	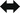 Work during eccentric exercise, MVC torque and CK activity between groups at all‐time points. 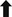 ROM in the third and fourth days after eccentric exercise in group 2 vs group 3. 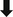 Muscle soreness (VAS scale) at 3‐days for the palpation of the upper arm in group 2 vs group 1 and 3; and at 4‐days for the extension of the elbow joint and vs group 3.
Nakhostin‐Roohi et al. (2016)	RCT, DB; 7 days intervention	Men, 25, 20, healthy and active	Running for 14 km	Group 1: 90 mg daily of curcumin for 7 days before the main exercise Group 2: placebo	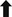 TAC before, immediately, 24, and 48 h after exercise in group 1 vs baseline as well as before and immediately after exercise vs group 2. 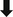 MDA‐TBARS in group 1 vs group 2 immediately after exercise 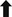 GSH immediately, 24, and 48 h after exercise vs pre‐exercise only in group 1.
Basham et al. (2019)	RCT, DB; 28 days intervention	Men, 21, 19, healthy and active participants	Eccentric muscle actions	Group 1: 1500 mg daily of curcumin (three capsules) Group 2: placebo	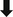 Muscle soreness (VAS scale) and CK in the group1 vs group 2. 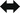 TAC, TNF‐α, and MDA between groups at all‐time points.
Drobnic et al. (2014)	RCT, SB; 4 days intervention	Men, 35, 19, healthy and moderately active	Downhill running	Group 1: 200 mg curcumin twice per day (from 48 h pre‐test to 24 h post‐test) Group 2: placebo	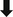 Muscle injury in MRT, MLT, PLT, and PRT in the group 1 vs group 2. 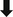 IL‐8 in the group 1 vs group 2. 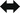 CK, hsCRP, MCP‐1, FRAP, CAT, GPx, pain score, and muscle histology for muscle injury and inflammation between groups.
Samadi et al. (2019)	quasi‐experimental; −	Men, 21, 45, healthy untrained	Resistance exercise included knee flexion with 70% of 1RM	Group 1: 1000 mg curcumin for 1 day (before trial) Group 2: 1000 mg/day curcumin for 5 days (before trial) Group 3: placebo	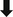 CK, LDH, and muscle pain in group 1 vs group 2 and 3. 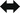 CK, LDH, and muscle pain between group 1 vs group 3.
Nicol et al. (2015)	RCT, DB, CO; two phases of 5 days separated by 14 days.	Men, 18–39, 17, healthy and light to moderate regular physical activity	Single‐leg jump performance	Group 1: 2.5 g curcumin twice per day (2 days before to 3 days after eccentric single‐leg press exercise) Group 2: placebo	Pain (VAS scale) and CK increased lower in the group 1 vs group 2. 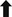 IL‐6 immediately and 24‐hr after exercise vs baseline and 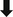 at 48‐hr after exercise vs immediately post‐exercise. 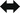 TNF‐α at all‐time points between groups.
McFarlin et al. (2016)	RCT, DB; 6 days intervention	Both, 20, 28, healthy	45° inclined seated dual leg press	Group 1: 400 mg curcumin (2 days before and 4 days after exercise) Group 2: placebo	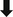 CK, TNF‐α, and IL‐8 in the group 1 vs group 2. 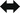 IL‐6, IL‐10, quadriceps muscle soreness, and ADL soreness between group 1 and 2.
Takahashi et al. (2014)	RCT, DB, CO; −	Men, 27, 10, healthy	Walking or running at 65% of VO_2max_ on a treadmill for 60 min	Group 1: single dose, 90 mg curcumin before exercise Group 2: double dose, 90 mg curcumin before and after exercise (180 mg) Group 3: placebo	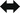 HR, RPE score, TBARs, GSSG, and SOD at all‐time points and between groups. 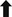 d‐ROMs and TRX after exercise in group 3, but not in the group 1 and 2. 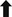 BAP after exercise in group 1 and 2; and GSH 2‐hr after exercise in group 1. 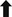 CAT after exercise in all groups, but 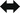 between groups. 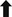 GPX and 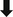 GR 2‐hr and immediately after exercise in group 3, respectively.
Tanabe, Chino, Ohnishi, et al. (2019)	Experimental, DB, CO; 7 days before and 7 days after exercise	Experiment 1: Men, 28.5, 10, healthy Experiment 2: Men, 29, 10, healthy	Eccentric exercise of the elbow flexors	Experiment 1: 7 days before exercise Group 1: 180 mg/day curcumin Group 2: placebo Experiment 2: 7 days after exercise Group 1: 180 mg/day curcumin Group 2: placebo	Experiment 1 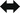 MVC torque, ROM, muscle soreness, CK activity, TNF‐α, d‐ROMs, and BAP concentration between group 1 and 2. 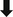 IL‐8 in the group 1 vs group 2 12‐h after exercise. Experiment 2 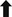 MVC torque and ROM, and 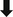 muscle soreness and CK activity after exercise in the group 1 vs group 2. 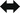 TNF‐α, IL‐8, d‐ROMs, and BAP between group 1 and 2.
Nakhostin‐Roohi et al. (2016)	RCT, DB, CO; −	Men, 25, 10, healthy	Quadriceps muscle exercise using a squat machine at under 50% 1RM	Group 1: 150 mg curcumin (immediately after exercise) Group 2: placebo	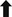 TAC in group 1 vs group 2 at 24‐h and 48‐h after exercise CK, ALT, and AST increased lower in the group 1 vs group 2 at 24‐h after exercise VAS increased lower in group 1 vs group 2 at 48‐h and 72‐h after exercise
Ali et al. (2019)	RCT; 4 weeks	Men; 19.7, 18, healthy and light to moderate intensity (3 times per week)	Endurance training using ergocycle with moderate intensity (50–70% HRMax)	Group 1: 1.1 g/day curcumin Group 2: placebo	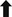 DHR (at week 2 and 4) and LC (at week 4) after exercise in group 1 vs baseline. 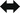 Leg power and jump height in group 1 vs baseline. 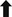 Leg power recovery in group 1 vs group 2. 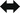 DHR, LC, and jump height recovery between group 1 and 2.

*Note*: The up arrows indicate the elevation, the down arrows indicate the reduction, and the Left–Right Arrow indicate no change.

Abbreviations: RCT, randomized control trial; DB, double blind; SB, single blind; CO, crossover; MVC, maximal voluntary contraction; CK, creatine kinase; ROM, range of motion; TNF‐α, tumor necrosis factor‐α; VAS, visual analog scale; IL‐6, Interleukin 6; ALT, alanine transaminase; AST, aspartate transaminase; TAC, total antioxidant capacity; MDA, malondialdehyde; TBARS, thiobarbituric acid‐reactive substance; PRT, posterior right thigh; MRT, medial right thigh; PLT, posterior left thigh; MLT, medial left thigh; hsCRP, high‐sensitivity C‐reactive protein; MCP‐1, monocyte chemoattractant protein‐1; FRAP, ferric reducing ability of plasma; CAT, catalase; LDH, lactate dehydrogenase; RPE, ratings of perceived exertion; GSSG, oxidized glutathione; TRX, thioredoxin; GR, glutathione reductase; SOD, superoxide dismutase; GPx, glutathione peroxidase; ADL, activities of daily living; HR, heart rate; BAP, biological antioxidant potential; DHR, decrease of heart rate; LC, lactate clearance; 1RM, one repeated maximum.

In a randomized, double‐blinded, placebo‐controlled trial by Basham et al., 19 healthy men who regularly engaged in aerobic activity were randomized to receive either 1500 mg curcumin (from three capsules), or placebo, for 28 days. The authors reported that curcumin did not affect the plasma total antioxidant capacity (TAC), TNF‐α, and MDA concentration in participants after exercise‐induced muscle damage. However, curcumin supplementation reduced muscle soreness and muscle damage as measured by a visual analog scale (VAS) and plasma CK concentrations, respectively, compared to these same parameters for participants that received placebo (Basham et al., 2019). Curcumin may reduce muscle pain by lowering prostaglandins (through the COX‐2 pathway) and inhibiting transient receptor potential ion channels (Kang et al., 2004; Yeon et al., 2010). As reported by Tanabe et al. (2015) curcumin (150 mg prior and 150 mg after exercise) did not affect TNF‐α, IL‐6, ROM, and muscle soreness (VAS scale) following eccentric exercise in participants, while maximal voluntary contraction (MVC) torque and recovery were higher and CK was lower in the curcumin‐treated group compared to those that received a placebo. A previous study by Tanabe et al. suggested that curcumin could be effective in shortening the recovery process. Ali et al. showed that 1.1 g/day of curcumin supplementation for 4 weeks, along with endurance training, significantly increased lactate clearance at week four and decreased heart rate at weeks two and four when compared to baseline values (Ali et al., 2019). Moreover, in a double‐blinded, randomized, controlled crossover trial by Nicol et al., 17 men who performed light to moderate regular physical activity were assigned to receive either curcumin (2.5 g b.i.d), or placebo, for 2.5 days before and 2.5 days after exercise. The authors observed that curcumin did not affect TNF‐α; although, the IL‐6 levels were increased immediately and at 48 h after exercise in the curcumin group when compared to baseline, but decreased 24 h after exercise compared to post‐exercise (immediately after the test). Additionally, measurement of quadriceps and gluteal skeletal muscle pain and function by VAS and single‐leg vertical squat jump showed a decrease in pain and increased performance, respectively, in the curcumin‐treated group (Nicol et al., 2015).

Mc Farlin et al. also designed a double‐blind, placebo‐controlled, clinical trial involving 28 participants that were randomly assigned to take either 400 mg curcumin, or placebo, daily from 2 days prior, to 4 days after, exercise. In disagreement with Nicol et al., curcumin significantly suppressed the levels of CK, TNF‐α (up to 4 days), and IL‐8 (up to 2 days) after exercise when compared to these same parameters in the participants who took the placebo, and there were no significant differences in either muscle soreness resulting from normal activities of daily living (ADL's), or IL‐6 and IL‐10, despite a similar response in IL‐8 and TNF‐α (McFarlin et al., 2016). The results regarding muscle soreness are in contradiction with the study by Basham et al., which is probably due to differences in assessment technique. Similar findings were reported by Nakhostin‐Roohi et al., who conducted two randomized controlled trials to demonstrate the effective timing of curcumin ingestion on oxidative stress and biomarkers of muscle soreness induced by exercise. The first study included 10 men that were divided into two groups and received either curcumin (150 mg), or placebo, immediately after heavy eccentric exercise. It was shown that the group receiving curcumin had higher total antioxidant capacity (TAC) and lower levels of markers of muscle damage including ALT, AST, and CK, as well as muscle soreness (VAS scale), at 48 h and 72 h after the test compared to the placebo (Nakhostin‐Roohi et al., 2016). The second study aimed to assess the effect of curcumin supplementation before exercise in active men. In this study, participants received either 90 mg curcumin, or placebo, for 7 days before the exercise protocol (14 km run). These authors found that the curcumin‐treated group exhibited improvement in TAC and glutathione, and a reduction in MDA‐thiobarbituric acid‐reactive substance (TBARS) after exercise (Roohi et al., 2017). Along these lines, a study by Takahashi et al. compared the efficacy of a single (before trial) and double (before and immediately after trial) dose of curcumin on oxidative stress resulting from running on a treadmill for 1 h. However, the study by Takahashi et al. did not show significant differences in thioredoxin‐1, derivatives of reactive oxygen metabolites (ROMs), SOD, GPx, TBARS, and oxidized glutathione, although catalase, glutathione, and the biological antioxidant potential (BAP) were increased in both the intervention (curcumin) group after exercise. These findings by Takahashi et al. were thought to be attributable to the ROS scavenging activity of curcumin and increased enzymatic and nonenzymatic antioxidant activity (Takahashi et al., 2014). Tanabe et al. also designed a cross‐over, double‐blind, placebo‐controlled, clinical trial in which 20 healthy men were assigned to receive either 180 mg curcumin, or placebo, daily in two parallel experiments including (1) 7 days before exercise (experiment 1, *n* = 10); (2) 7 days after exercise (experiment 2, *n* = 10). The results showed that curcumin did not affect TNF‐α, IL‐8, joint range of motion (ROM), and the BAP compared to the placebo when consumption of curcumin occurred after exercise (experiment 2), whereas, it was able to mediate a reduction in elevated IL‐8 levels 12‐h after exercise when it was consumed before exercise (experiment 1). Also, this study indicated a reduction in CK and muscle soreness and an increase in joint range of motion and isometric maximal voluntary contraction (MVC) torque in experiment 2 only (Tanabe, Chino, Ohnishi, et al., 2019). A similar study by Tanabe et al. evaluated the effect of curcumin on muscle damage markers when taken before and after exercise. In disagreement with the previous study, 150 mg/kg of curcumin did not impact joint range of motion and MVC torque in both intervention (curcumin) groups (before and after the trial) compared to placebo. However, these same authors reported that muscle soreness (VAS scale) decreased and joint range of motion increased only when curcumin was consumed for 4 days after exercise compared to curcumin consumption for 7 days before exercise and when each of these same parameters were compared to corresponding measurements obtained in subjects that ingested the placebo. Altogether, these findings suggested that a decrease in the production of pain mediators and the sensation of pain resulting from curcumin consumption after exercise may be the reason for reduced muscle soreness when compared to curcumin consumption before exercise (Tanabe, Chino, Sagayama, et al., 2019).

Moreover, in another randomized, placebo‐controlled, single‐blind pilot trial, 19 healthy males were randomly assigned to receive either 200 mg curcumin (b.i.d), or placebo (b.i.d), for 4 days (initiated from 48‐h before to 24‐h after moderate aerobic activity). The results showed a lower level of IL‐8 in the curcumin group compared to the subjects in the placebo group; however, despite a slight increase in high‐sensitivity C‐reactive protein, monocyte chemoattractant protein‐1, catalase, GPx, ferric reducing ability of plasma, and CK (as a marker of muscle damage) in the curcumin group compared to the placebo, none of these measurements reached statistical significance between the two groups. In addition, muscle damage was assessed using both MRI and histological analyses 48‐h after the exercise. Results from MRI showed a reduction in muscle damage in the posterior or medial compartment of the right and left thigh in the treatment group compared to the placebo, while immunohistochemical staining did not show any change in sarcolemmal damage between the two groups (*n* = 4 for treated‐group, *n* = 5 for the placebo group). These authors attributed the lack of statistical difference in MRI and immunohistochemcial results to the short duration of the study and the small number of samples used (Drobnic et al., 2014). Based on the above studies, curcumin supplementation appears to be potentially effective in reducing muscle pain, muscle enzyme activity, and biomarkers of inflammation and oxidative stress caused by exercise, especially when curcumin is used during the recovery period as opposed to ingestion prior to the exercise. It is suggested that the lack of significant differences in some of these studies may potentially be attributed to the type and intensity of exercise, as well as the small number of samples or subjects utilized.

The duration of administration and the dose of curcumin consumed appear to be important factors for determining the efficacy of curcumin on exercise performance. In a quasi‐experimental study, Samadi et al. determined the efficacy of curcumin at a daily dose of 1000 mg for 1 and 5 days before an eccentric exercise session to assess exercise‐induced muscle damage. The authors reported that curcumin supplementation effectively reduced muscle pain, CK, and lactate dehydrogenase for a longer period of time when the 5‐day pre‐treatment regimen of curcumin was used compared to the 1‐day pretreatment regimen (Samadi et al., 2019). Also, with regard to exercise, it is important to consider the time that curcumin is used in order to optimize efficacy. Results from numerous studies have demonstrated that plasma curcumin levels remain significantly elevated when curcumin ingestion occurs after exercise, whereas there is typically no curcumin detectable in the plasma after exercise when curcumin ingestion occurs before the exercise (Takahashi et al., 2014; Tanabe et al., 2015; Tanabe, Chino, Sagayama, et al., 2019).

The effect of curcumin on muscle pain was assessed in nine studies included in this review (Basham et al., 2019; Drobnic et al., 2014; Jäger et al., 2019; McFarlin et al., 2016; Nicol et al., 2015; Samadi et al., 2019; Tanabe et al., 2015; Tanabe, Chino, Ohnishi, et al., 2019; Tanabe, Chino, Sagayama, et al., 2019). Five of them indicated that curcumin supplementation (180–2500 mg) was able to reduce muscle soreness (Basham et al., 2019; Nicol et al., 2015; Samadi et al., 2019; Tanabe, Chino, Ohnishi, et al., 2019; Tanabe, Chino, Sagayama, et al., 2019), while four other studies showed nonsignificant effect of curcumin on muscle soreness (Drobnic et al., 2014; Jäger et al., 2019;McFarlin et al., 2016; Tanabe et al., 2015). In terms of the effect of curcumin supplementation (150–2500 mg) on muscle damage after exercise, eight studies also showed a decrease in muscle damage as determined by enzyme production (Basham et al., 2019; McFarlin et al., 2016; Nakhostin‐Roohi et al., 2016; Nicol et al., 2015; Samadi et al., 2019; Tanabe et al., 2015; Tanabe, Chino, Ohnishi, et al., 2019), or MRI results (Drobnic et al., 2014), and only one study did not show the effect of curcumin on muscle damage (Tanabe, Chino, Sagayama, et al., 2019). Thus, these findings suggest that curcumin supplementation may be effective in reducing muscle damage after exercise. While it is difficult to find consensus on whether curcumin reduces muscle soreness, this may be due to differences in the perception of pain and the way it is quantified.

In theory, increased muscle damage and muscle soreness after exercise may be attributable to inflammation and oxidative stress (Aoi et al., 2004; Tauler et al., 2002). Several studies have demonstrated that increased muscle injury caused by exercise was modified by curcumin supplementation, which also led to lower levels of inflammation and biomarkers of oxidative stress (TNF‐α, IL‐8, and TAC) (Drobnic et al., 2014; McFarlin et al., 2016; Nakhostin‐Roohi et al., 2016). Therefore, the inhibitory effects of curcumin on muscle injury may be due to its modulating effects on inflammation and oxidative stress, as confirmed in several studies, which demonstrated that curcumin supplementation decreased the activity of myeloperoxidase (MPO) and the expression of TNF‐α, IL‐6, and IL‐1β, as well as inhibited TLR4‐mediated NF‐KB signaling pathways (Das & Vinayak, 2014; Fu et al., 2014). Again, as mentioned previously, it is difficult to achieve consensus on the effect of curcumin supplementation to reduce inflammation and oxidative stress after exercise. While some studies have shown that curcumin can exert anti‐oxidant effects (Nakhostin‐Roohi et al., 2016; Roohi et al., 2017; Takahashi et al., 2014) and decrease the levels of biomarkers associated with inflammation (e.g., IL‐6 (Nicol et al., 2015), IL‐8 (Drobnic et al., 2014; McFarlin et al., 2016; Tanabe, Chino, Ohnishi, et al., 2019), and TNF‐α (McFarlin et al., 2016)), other studies failed to show any change in oxidative stress (Basham et al., 2019; Drobnic et al., 2014) and inflammatory biomarkers (i.e., IL‐6 (McFarlin et al., 2016; Tanabe et al., 2015) and TNF‐α (Basham et al., 2019; Nicol et al., 2015; Tanabe et al., 2015; Tanabe, Chino, Ohnishi, et al., 2019)) with curcumin treatment. There have been other alternative explanations advanced regarding the possible mechanisms responsible for the reduction in muscle soreness obtained with curcumin supplementation. One such premise is that curcumin decreases mediators induced by cyclooxygenase‐2, including prostaglandin E2, histamine, bradykinin, and serotonin through suppression of its gene expression (Hatcher et al., 2008; Tanabe, Chino, Sagayama, et al., 2019).

Five studies evaluated the effect of curcumin on maximal voluntary contraction (MVC), joint range of motion (ROM) (Takahashi et al., 2014; Tanabe et al., 2015; Tanabe, Chino, Ohnishi, et al., 2019; Tanabe, Chino, Sagayama, et al., 2019), and muscle function via isokinetic dynamometry (Jäger et al., 2019) as metrics for determining the effects of curcumin on the performance of exercise. Three studies performed by Tanabe, Chino, Ohnishi, et al. (2019), Tanabe, Chino, Sagayama, et al. (2019), Tanabe et al. (2015) showed that curcumin (180 mg after, and 300 mg before and after exercise) increased MVC and ROM; however, Takahashi et al. (2014) did not show a significant change in ROM. As it pertains to muscle function measurements using isokinetic dynamometry, Jäger et al. (2019) reported that curcumin (200 mg) was able to prevent a reduction in isokinetic peak extension and flexion torque. This difference seems to be due to the difference in supplementation time compared to exercise and study duration. From the point of view of when to take a curcumin supplement in relation to the time at which the exercise occurs, it appears to be more effective when taken pre‐ and post‐workout.

Finally, as mentioned previously, it is essential to consider the intensity, duration, and type of exercise, as well as the appropriate dose of curcumin, when evaluating the positive effects of curcumin on exercise performance. Numerous studies have shown that curcumin and exercise both have a hormetic effect, which is defined as a biphasic response to exposure. Interpreted in the context of curcumin supplementation and exercise, this means that curcumin at high and low doses (which corresponds to high and low plasma concentrations of curcumin, respectively) may have a negative effect on exercise. In our previous study, we provided a comprehensive review of the hormetic behavior of curcumin under different conditions (Moghaddam et al., 2019). In terms of exercise, Radak et al. reported that participants with low‐ and high‐intensity exercise programs were at a higher risk for oxidative stress, while those participants that engaged in regular exercise of moderate intensity had lower levels of oxidative stress (Radak et al., 2008). On the other hand, Ristow et al. indicated that an increase in ROS within mitochondria may induce an adaptive response that increases resistance to oxidative stress and, in addition, reduces long‐term stress. This adaptive response of mitochondria to increased ROS, which is known as the “mitochondrial synthesis” hypothesis, has been suggested to be responsible for the progressive health gains and lifespan‐extending benefits of regular exercise (Ristow & Zarse, 2010). It is not clear at the present time whether this process/hypothesis is applicable to humans, since several epidemiological studies have suggested that supplementation with some antioxidants may increase the prevalence of disease in humans (Bjelakovic et al., 2007). Thus, additional studies are needed to determine whether supplementation with antioxidants, such as curcumin, may confer beneficial health effects, or may induce adverse effects on normal cell function.

## 2 CONCLUSIONS

We reviewed the existing evidence for the effects of curcumin administration on exercise performance in preclinical studies and clinical trials. The preclinical studies in this review strongly support the beneficial effects of curcumin administration in reducing exercise‐induced complications, including muscle damage and muscle pain, which are thought to occur due to a reduction in inflammation and oxidative stress. Accordingly, this results in better exercise performance and a faster recovery. While the clinical evidence has shown promising results for curcumin supplementation to mediate a reduction in muscle damage and pain, inflammation, and oxidative stress, it is not overwhelmingly convincing. In general, it appears that curcumin supplementation can have positive effects on exercise performance and recovery, muscle damage and pain, inflammation, and oxidative stress, especially when taken after the exercise. However, there is still a need to further examine factors such as bioavailability, dosage, frequency of curcumin administration, time of curcumin supplementation relative to the time at which the exercise occurs, and the type and intensity of the exercise, as well as the age and gender of the athlete. Finally, curcumin, via its antioxidant properties, may be effective in reducing cellular oxidative stress, especially in mitochondria over the long term, as well as positively impacting exercise endurance, strength, and recovery to improve overall health.

## CONFLICT OF INTEREST

None.

## Data Availability

There is no primary data associated with this manuscript.
